# Predictors of pelvic pain in a general urology clinic population

**DOI:** 10.1002/bco2.262

**Published:** 2023-07-01

**Authors:** Grace Prillaman, Jacqueline Zillioux, Haerin Beller, Clinton Yeaman, David Rapp

**Affiliations:** ^1^ University of Virginia School of Medicine Charlottesville Virginia USA; ^2^ Department Urology University of Virginia Charlottesville Virginia USA

**Keywords:** chronic pelvic pain, depression, fibromyalgia, irritable bowel syndrome, overactive bladder

## Abstract

**Objectives:**

To assess the prevalence and predictors of chronic pelvic pain in a general urology population presenting for evaluation of unrelated non‐painful complaints.

Generalized pelvic pain is estimated to afflict between 6% and 26% of women and is often multifactorial in aetiology. A paucity of prospective research exists to characterize chronic pelvic pain patterns and to understand related predictors.

**Materials and Methods:**

This is a prospective, cross‐sectional survey‐based study of female patients presenting to a general urology clinic over a 10‐month period (7/2018–5/2019). Patients completed a 32‐item survey with questions pertaining to demographics, comorbidities and chronic pelvic pain characteristics. Comparison tests (chi‐squared, Fisher's exact) and stepwise multivariable logistic modelling were performed to assess for predictors of chronic pelvic pain.

**Results:**

A total of 181 women completed the survey, with a mean age of 56 years. Overall, 75 (41%) women reported chronic pelvic pain. Those with chronic pelvic pain were younger compared to those without (52 vs 59 years, *p* = 0.001). Univariable logistic regression analysis identified BMI, depression, fibromyalgia, overactive bladder and any bowel symptoms as possible positive predictors of chronic pelvic pain. Final best‐fit multivariable model found overactive bladder, fibromyalgia and presence of bowel symptoms as independent positive predictors of chronic pelvic pain.

**Conclusions:**

Our study is one of the few studies that has prospectively analysed chronic pelvic pain and its predictors. The present study identified significant associations with overactive bladder, fibromyalgia and bowel symptoms. Further research is needed to better understand the aetiologies of chronic pelvic pain and the possible relationship with identified clinical predictors.

## 1 INTRODUCTION

Chronic pelvic pain (CPP) affects a large proportion of the population, yet little research has been directed at determining the predictors and comorbidities of this condition. CPP is defined as pain lasting typically 6 or more months that is perceived to be originating from within the pelvis and is often associated with gynaecologic, bowel, lower urinary tract, sexual symptoms or negative cognitive and emotional effects.^1^ Studies have estimated that the prevalence of CPP ranges from 2% to upwards of 40% of women.^2^ The relationship between CPP and these comorbidities is poorly defined, and there is a scarcity of data characterizing CPP patterns and its related predictors. Prior studies have added to our understanding of CPP by assessing the prevalence and phenotypes of CPP^3^; we aim to expand upon this previous research and prospectively analyse the association between CPP and its predictors, specifically focusing on overactive bladder (OAB), fibromyalgia, depression and irritable bowel syndrome (IBS).

## 2 MATERIALS AND METHODS

A prospective, survey‐based, single‐institution study of female patients presenting to a general urology clinic was conducted over a 10‐month period from 7/2018 to 5/2019. Participants were recruited during clinic registration by a study coordinator. Written informed consent was obtained prior to participation in the study. Patients who were less than 18 years old, prisoners or pregnant were excluded. Additionally, patients presenting to clinic with acute pain‐related complaints, such as flank pain, acute pelvic pain or interstitial cystitis, were excluded in order to specifically address CPP. This study was approved by the University of Virginia Institutional Review Board (protocol # 20503). All enrolled participants completed a 23‐item survey with questions relating to demographics, past medical history and pelvic pain symptoms. The questionnaire assessed pain location using detailed anatomic figures as well as through written multiple‐choice questions. Additional multiple‐choice and Likert scale questions addressed pain quality, severity, location and the impact on the patient's life. Pain severity was assessed on a scale of 1–10 with ‘10’ being defined as *terrible pain* on average over the past month. Pain location was assessed using a multiple‐choice format that allowed patients to select all of the areas they experience pain (urethra, vagina, buttocks, bladder/lower abdomen, upper abdomen, hips, thighs, lower back) well as a diagram that allowed them to mark on an illustration of a body where they experience pain. Pain quality was assessed by participants self‐reporting pain to be sharp/stabbing, achy/dull, tingling/numbness or pain to touch. Chart review was completed to gather other demographic and comorbidity information not covered in the survey.

### 2.1 Statistical analysis

Data analysis was performed using the R programming language (Version 4.4.1). Demographic data and presenting concerns were collected. Logistic regression models were constructed with the outcome variable indicating the occurrence of CPP and predictor variables being posited predictors of CPP. These included demographics and comorbid conditions. Univariable logistic regression modelling followed by a final multivariable logistic regression model were conducted. All tests were performed with α = 0.05.

## 3 RESULTS

A total of 187 women completed the survey, with a mean age of 56 years. Six patients were excluded from analysis due to chief complaint of interstitial cystitis or acute pelvic pain that was not elicited during registration. Of the 181 included patients, 75 (41%) reported experiencing CPP. The chief complaints of patients presenting to clinic are represented in Table 1 with stones (19%) and ‘other’ (19%) being the most common among patients reporting CPP, while urinary incontinence (32%) was the most common among those without pain. Univariable logistic regression modelling found that depression and a higher body mass index (BMI) were a risk factors for CPP (OR = 2.83, *p* = 0.001; OR = 1.05, *p* = 0.01). Multivariable logistic regression model found that fibromyalgia, OAB and bowel symptoms were independent positive predictors of CPP, as seen in Table 2 (OR = 4.16, *p* = 0.02; OR = 2.65, *p* = 0.005; OR = 3.46, *p* < 0.001). Older age was found to be protective against CPP (OR = 0.98, *p* = 0.001).

**TABLE 1:** Demographics.

Chief complaint	No pain (*n* = 106)	CPP (*n* = 75)
Haematuria	9 (8.6%)	3 (4.1%)
Stones	20 (19%)	15 (20%)
Retention	4 (3.8%)	1 (1.4%)
UI	16 (15%)	24 (32%)
rUTI	9 (8.6%)	11 (15%)
POP	2 (1.9%)	2 (2.7%)
UCC	10 (15%)	5 (5.4%)
RCC	15 (14%)	4 (5.4%)
Other	20 (19%)	10 (14%)

Abbreviations: CPP, chronic pelvic pain; POP, pelvic organ prolapse; RCC, renal cell carcinoma; rUTI, recurrent urinary tract infections; UCC, urothelial carcinoma; UI, urinary incontinence.

**TABLE 2:** Predictors of chronic pelvic pain.

	Unadjusted 95% CI			Adjusted 95% CI		
	OR	*p*		OR	*p*	
Age (year)	0.97	0.001	*p* < 0.01	0.98	0.03	*p* < 0.05,
BMI (kg/m^2^)	1.05	0.01	*p* < 0.05			
Smoker	1.51	0.32				
Diabetes	0.91	0.78				
Depression	2.83	0.001	*p* < 0.01			
Fibromyalgia	5.80	0.001	*p* < 0.01	4.16	0.02	*p* < 0.05
Overactive bladder	2.51	0.003	*p* < 0.01	2.65	0.005	*p* < 0.01
Endometriosis	1.72	0.35				
OB complication	0.99	0.97				
Pregnancies	1.15	0.19				
Surgical history						
Abdominal	1.01	0.97				
Pelvic	0.80	0.49				
IBS	2.73	0.01	*p* < 0.05			
GI symptoms (any)	4.30	<0.001	*p* < 0.001	3.46	<0.001	*p* < 0.001
Bloating	2.69	0.003	*p* < 0.01			
Constipation	2.69	0.002	*p* < 0.01			
Diarrhoea	2.41	0.007	*p* < 0.01			
Bowel pain	6.38	<0.001	*p* < 0.001			

*Note*: OB complication, obstetric complication (any of forceps's delivery, caesarean section, post‐partum haemorrhage, episiotomy); bowel symptoms, any of diarrhoea, constipation, bloating or bowel pain.

Abbreviation: IBS, irritable bowel syndrome.

## 4 DISCUSSION

The present study found that fibromyalgia, OAB, bowel symptoms and younger age were independent predictors of CPP, while higher BMI and the presence of depression were found to be possible predictors of CPP. The association between these predicators and CPP is significant as the symptoms of fibromyalgia, OAB and bowel symptoms may distract from the true cause of CPP, making diagnosis and treatment more difficult; additionally, these conditions may play a causal role in CPP and more increase the severity of the condition. Understanding that CPP, fibromyalgia, OAB and bowel symptoms often co‐occur and may be involved the in pathogenesis of CPP is critical to avoiding a delay in diagnosis and appropriately treating these patients.

### 4.1 Fibromyalgia

As one of the few studies to prospectively evaluate CPP and its predictors, the present study's finding that fibromyalgia is an independent predictor of female CPP is in line with prior research demonstrating that patients with CPP have a higher incidence of fibromyalgia (4%–31%) compared to the general population.^4^ It has been hypothesized that CPP and fibromyalgia both occur to some degree through the process of central sensitization. Central sensitization occurs when chronic pain and noxious stimuli lead to alterations in the central nervous system causing hyperalgesia and a distorted or amplified perception of pain.^5^ Fibromyalgia is the most common central sensitization syndrome and increasing severity of fibromyalgia has been correlated with increasing severity of CPP in patients in which these conditions co‐occur.^6^, ^7^ Although fibromyalgia and CPP often present together, they are considered separate pain syndromes. While the aetiology of CPP is unclear, it is most often attributed to myofascial dysfunction with upwards of 50%–90% of CPP patients presenting with pain from a musculoskeletal origin.^8^ CPP has also been associated with the presence of myofascial trigger points on exam, with more trigger points corresponding to higher levels of pain sensitization; these trigger points differ from the tender points characteristic of fibromyalgia.^9^ While fibromyalgia and CPP are pain syndromes that occur together at high rates, differentiating between the two syndromes is critical to providing the proper treatment to patients.

### 4.2 OAB

OAB was found to be an independent predictor of CPP in this study population. Prior work has estimated that 24% of patients with CPP also experience urinary symptoms, such as urgency urinary incontinence (UUI), stress urinary incontinence (SUI) and other lower urinary tract symptoms (LUTS).^10^, ^11^ Central sensitization, as discussed above in relation to CPP, has also been implicated in the development of OAB and increased incidence of LUTs.^11^, ^12^ Furthermore, increased OAB symptoms are thought to cause increased pain intensity and somatic symptom burden in patients with any type of pain.^11^ For CPP specifically, OAB has been posited to increase pain by causing inactivity, a known risk factor for pain, due to the nature of the symptoms of OAB.^13^ CPP and OAB are further connected by their shared relationship to myofascial pelvic floor dysfunction and treatment with pelvic floor physical therapy.^14^ While pelvic floor physical therapy has long been considered first‐line therapy for pelvic floor pain, it is also used for the management of OAB.^15^ Initiation of pelvic floor activation, as is worked on during pelvic floor physical therapy, has been shown to abort errant detrusor contractions that are responsible for OAB and UUI.^16^

### 4.3 Bowel symptoms

In this study, bowel symptoms, which were defined as self‐reported constipation, bloating, diarrhoea or bowel pain within the last month on the survey, were found to be an independent predictor of CPP. It has been estimated that over 38% of women with CPP have concurrent IBS symptoms and upwards of 37% of CPP is thought to be attributable to a gastrointestinal aetiology with IBS being the most common diagnosis.^10^, ^17^ Despite this finding and their common comorbidity, there are also data supporting IBS and CPP being separate entities.^18^ Both CPP and IBS have been independently associated with higher incidence of mood disorders, such as depression and anxiety, and a history of abuse; however, the incidence of mood disorders and a history of abuse is higher among those with CPP and IBS compared to IBS alone.^19^, ^20^, ^21^ A history of physical or sexual abuse is thought to cause somatic memory of pain leading to visceral hypersensitivity that can cause symptoms of CPP and IBS.^22^ Regardless as to whether CPP is caused by IBS or the two are separate entities, recognizing their pattern of common comorbidity is critical to providing appropriate care to patients. Patients presenting with symptoms of either CPP or IBS should be screened for symptoms other in order to achieve comprehensive symptom management in these patients.

### 4.4 Depression

The finding in the present study that depression serves as a possible positive predicator of CPP builds upon prior studies that have found that patients with CPP have a significantly higher incidence of depression compared to those without pain.^23^ Beyond this, it has been found that functional impairment in CPP may be more related to depressive and anxiety symptoms than pain severity itself.^24^ Patients with CPP are also more likely to have experienced sexual, physical, verbal and emotional abuse at some point during their life.^25^ This finding interconnects not only depression and CPP but also IBS and CPP, as noted above.^24^ It has been postulated that depressive symptoms are thought to lead to and worsen pain by causing inactivity, a known risk factor for pain.^13^ These findings highlight the multimodal nature of CPP and the need to address both depressive symptoms and pain in order to achieve adequate symptom control in these patients.

### 4.5 Study limitations and conclusions

The use of a non‐validated questionnaire and self‐report surveys introduced inherent biases and limitations to the present study. In addition, the focus of this study on urology patients at a single institution may limit its ability to be generalize the results to the broader population. Furthermore, excluding patients with chief concerns of acute pain in order to focus on CPP limited our sample size and may have excluded additional patient with CPP. Finally, this study did not include any physical exam findings in the assessment of patients' symptoms, which limits the conclusions that can be drawn regarding the nature and origin of patient's pain.

CPP afflicts a large proportion of the female population. This study demonstrated through prospective research a predictive relationship of fibromyalgia, depression, OAB and bowel symptoms to CPP. Further research is needed to elucidate the causality out these relationships. A better understanding of the interplay between these conditions will allow physicians to provide more comprehensive and effective care to their patients.

## AUTHOR CONTRIBUTIONS

*Conceptualization*: David Rapp, Haerin Beller and Jacqueline Zillioux. *Methodology*: Rapp, Haerin Beller, Jacqueline Zillioux and Clinton Yeaman. *Formal analysis*: Clinton Yeaman, Haerin Beller and Jacqueline Zillioux. *Investigation*: Clinton Yeaman and Grace Prillaman. *Data curation*: Clinton Yeaman and Haerin Beller, Jacqueline Zillioux. *Writing—original draft preparation*: Grace Prillaman. *Writing—review and editing*: Grace Prillaman, Haerin Beller and Jacqueline Zilliioux. All authors have read and agreed to the published version of the manuscript.

## CONFLICT OF INTEREST STATEMENT

The authors declare no conflict of interest.

## Supporting information


**Data S1.** Supporting Information.
