# Supplementary material for: Predictors of pelvic pain in a general urology clinic population
Source: BJUI Compass. 2023 Jul 1;4(6):668–72. doi: 10.1002/bco2.262 (PMC10560618; doi:10.1002/bco2.262)
Supplement: Supplementary file 1 — Data S1. Supporting Information. [file BCO2-4-668-s001.pdf]

WOMENS HEALTH SURVEY

SECTION 1. GENERAL INFORMATION

1. What is your age?

2. What is your weight?

3. What is your height?

4. What is your race?

- ◯ Caucasian
- ◯ Native American
- ◯ Asian
- ◯ African American
- ◯ Hispanic
- ◯ Other

5. What is your activity level?

- ◯ Sedentary (minimally active, minimal exercise)
- ◯ Mildly Active (some light exercise about 1x/week)
- ◯ Moderately Active (active job or moderate exercise several times weekly)
- ◯ Very Active (active job or frequent exercise)

6. Education

- ◯ High School (or GED)
- ◯ 2-year college
- ◯ 4-year college
- ◯ Graduate or Professional School

7. Annual Income

- ◯ Less than $50,000
- ◯ $50,000-$100,000
- ◯ More than $100,000

8. How many children do you have?

9. Where there any difficulties during pregnancy or delivery?

- ◯ Episiotomy/vaginal laceration
- ◯ Forceps or Vacuum delivery
- ◯ C-section
- ◯ Post-partum hemorrhage/bleeding

10. Please select any medical problems your have:

- ◯ Diabetes
- ◯ Depression
- ◯ Cancer
- ◯ Blood Pressure
- ◯ Fibromyalgia
- ◯ Smoking
- ◯ Cholesterol
- ◯ Irritable Bowel Syndrome
- ◯ Endometriosis

11. Have you ever had surgery for incontinence (e.g. bladder sling?)

- ◯ Yes
- ◯ No

12. Have you ever had surgery for vaginal prolapse (e.g. bladder tack?)

- ◯ Yes
- ◯ No

13. In the past month, have your experienced:

- ◯ Constipation
- ◯ Bloating
- ◯ Diarrhea
- ◯ Bowel Pain

WOMENS HEALTH SURVEY

SECTION 2. GENERAL PELVIC PAIN

14. Do you experience pelvic pain?

◯ Yes ◯ No

If YES, please continue with remaining questions
If NO, please go to SECTION 3

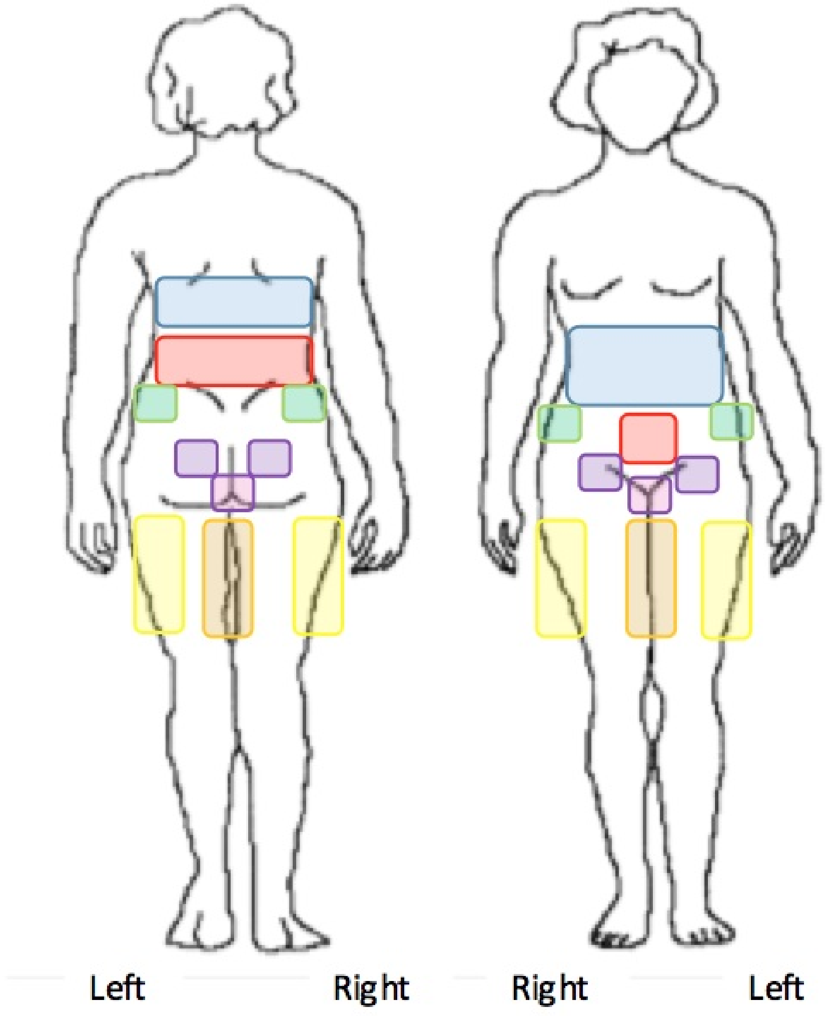

Please place an X over the shaded areas in which you feel pain

15. Please select all the areas you feel pain:

- ◯ Urethra
- ◯ Vagina
- ◯ Bladder/Lower abdomen
- ◯ Upper abdomen
- ◯ Hips
- ◯ Thighs
- ◯ Buttocks
- ◯ Lower back

16. When did you first notice the start of your pain symptoms?

17. How often have you had pain in these areas in the last month?

- ◯ Never
- ◯ Rarely
- ◯ Sometimes
- ◯ Often
- ◯ Usually
- ◯ Always

18. Which number describes your AVERAGE pain over the last month?

(No Pain) 0 1 2 3 4 5 6 7 8 9 10 (Terrible Pain)

19. Please check all the situations when you have pain:

- ◯ During exercise
- ◯ With walking
- ◯ With sitting
- ◯ All the time

20. Which of these best describe how your pain feels?

- ◯ Sharp/Stabbing
- ◯ Dull/Aching
- ◯ Tingling/Numbness
- ◯ Pain caused by touch

21. Over the last month, how much have your symptoms kept you from doing the things you would usually do?

- ◯ None
- ◯ Only a little
- ◯ Som e
- ◯ A lot

22. If you were to spend the rest of your life with your symptoms just the way they are, how would you feel?

- ◯ Delighted
- ◯ Mostly Satisfied
- ◯ Mixed (equally satisfied and dissatisfied)
- ◯ Unhappy
- ◯ Terrible

WOMENS HEALTH SURVEY

SECTION 3. PAIN WITH SEX

23. Are you sexually active?

- ◯ Yes
- ◯ No

24. If Yes, frequency of sexual activity

- ◯ Less than 1x/month
- ◯ 1-2/month
- ◯ 1-2/week
- ◯ More than 2x/week

25. If No, are there things that inhibit your sexual activity?

- ◯ Lack of interest in spouse
- ◯ Lack of interest by spouse
- ◯ Fear of pain
- ◯ Fear of poor performance
- ◯ Other: ______________________

26. Do you sometimes have pain with intercourse?

- ◯ Yes
- ◯ No

If YES, please continue with remaining questions
If NO, stop survey here

27. At what age did you start having pain with intercourse?

[ ]

28. Please select all the areas you feel pain with sex:

- ◯ Outside: Labia
- ◯ Outside: Clitoris
- ◯ Entrance to vagina
- ◯ Vagina: upper third
- ◯ Vagina: middle third
- ◯ Vagina: lower third
- ◯ Vagina: front wall
- ◯ Vagina: back wall
- ◯ Vagina: right side
- ◯ Vagina: left side

29. Which of these best describe how your pain feels?

- ◯ Sharp/Stabbing
- ◯ Tingling/Numbness
- ◯ Dull/Aching
- ◯ Pain caused by touch

30. Over the past month, how SATISFIED are you with your overall sex life?

- ◯ Very satisfied
- ◯ Moderately satisfied
- ◯ Moderately dissatisfied
- ◯ Very dissatisfie d

31. Over the past 4 month, how would you rate your LEVEL of pain with sex?

- ◯ Very high
- ◯ Moderate
- ◯ None
- ◯ High
- ◯ Low

32. Over the past 4 weeks, how often did you experience pain with sex?

- ◯ Never
- ◯ Sometimes (about half the time)
- ◯ Almost always
- ◯ A few times (less than half the time)
- ◯ Most times (more than half the time
